# Case Report: A case of severe infection caused by *Clostridium septicum*: a literature review

**DOI:** 10.3389/fmed.2026.1793472

**Published:** 2026-03-30

**Authors:** Jing Liu, Lu Wang, Yuyun Wang, You Zhang, Yuanqing Qu, Yuan Liu

**Affiliations:** 1 Department of Laboratory Medicine, The 943th Hospital of Joint Logistic Support Force of Chinese People’s Liberation Army, Wuwei, Gansu, China; 2 Department of Laboratory Medicine, The General Hospital of Western Theater Command of the People’s Liberation Army (PLA), Chengdu, Sichuan, China

**Keywords:** 16S rRNA gene sequencing, *Clostridium septicum*, MALDI-TOF MS, tetanus-like symptoms, traumatic infection

## Abstract

*Clostridium septicum*, an anaerobic spore-forming Gram-positive bacillus, is a rare opportunistic pathogen that causes severe infections with rapid progression and high mortality. Trauma with unmanaged contaminated wounds is a major risk factor, and atypical presentations such as tetanus-like neuromuscular symptoms often lead to misdiagnosis. Herein, we report a 72-year-old male farmer with *C. septicum* and *Enterobacter cloacae* co-infection following a traumatic toe laceration, presenting with trismus, limited mouth opening, and right toe gangrene. This case highlights the diagnostic challenges of *C. septicum* infection mimicking tetanus and the importance of comprehensive etiological testing and multidisciplinary management. We also review relevant literature to provide insights for clinical practice.

## 1 Introduction

*Clostridium septicum*, an anaerobic spore-forming Gram-positive bacillus, is a rare yet life-threatening opportunistic pathogen, causing infections with rapid progression and high mortality (1, 2). Clinical studies have documented that *C. septicum* infection is closely linked to contaminated traumatic wounds, underlying gastrointestinal malignancies, and immunocompromised conditions (2–4). Notably, approximately 50–60% of patients with *C. septicum* bacteremia present concurrent gastrointestinal abnormalities, which enable bacterial invasion by disrupting the mucosal barrier (5). A distinct clinical challenge is the atypical manifestation of tetanus-like neuromuscular symptoms, which often leads to misdiagnosis and delayed intervention (2, 6, 7). Herein, we report a 72-year-old male farmer with *C. septicum* and *Enterobacter cloacae* coinfection following unmanaged traumatic toe laceration, who presented with trismus, limited mouth opening, and right toe gangrene. This case adds to the existing literature on trauma-associated *C. septicum* infections (3, 5, 8, 9) and provides practical insights into differential diagnosis and multidisciplinary management. Furthermore, we review relevant clinical studies and expert consensus to discuss the diagnostic and therapeutic strategies for this disease, aligning with current clinical practice guidelines (1, 3, 9).

## 2 Case presentation

**FIGURE 1:**
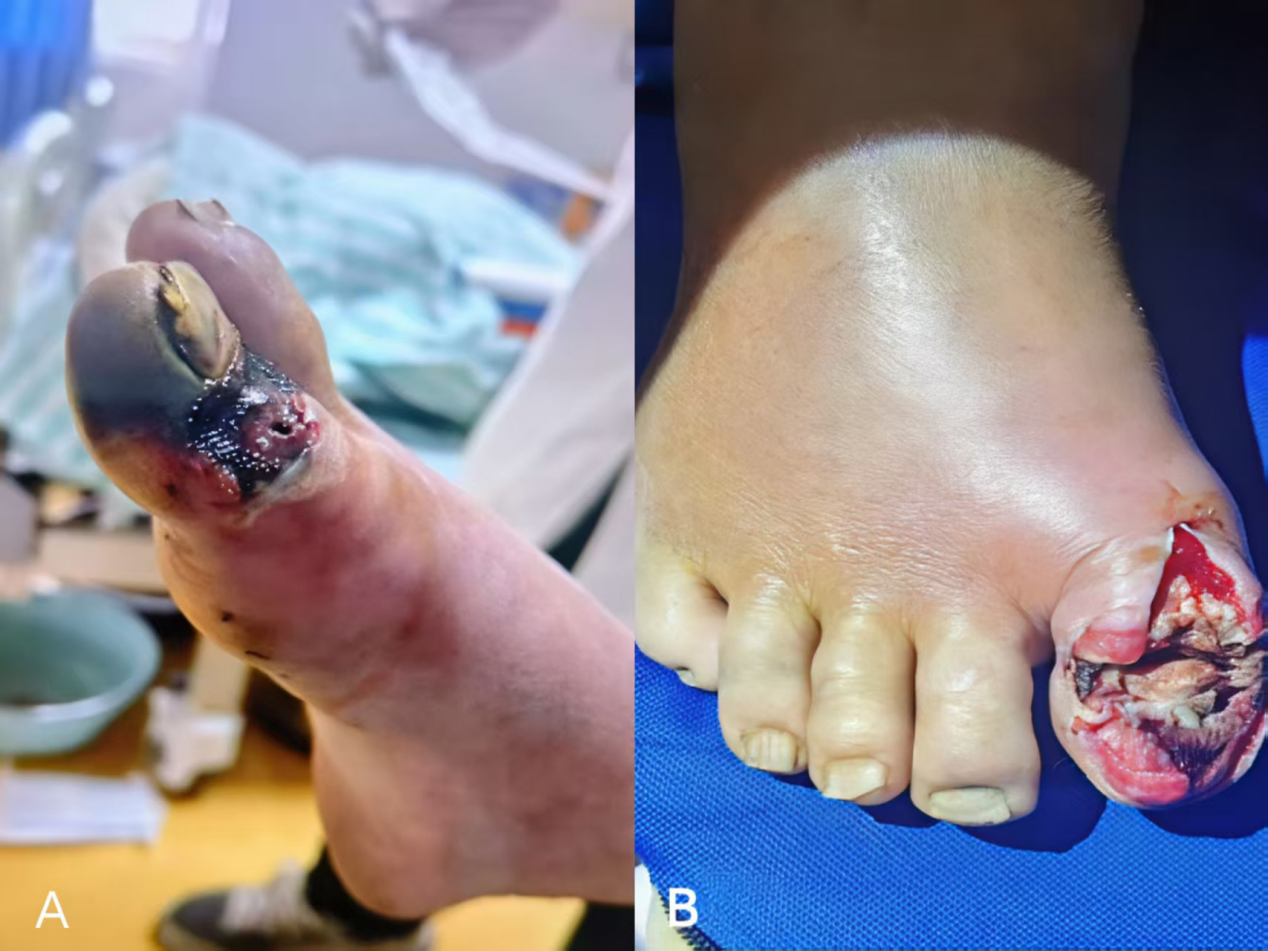
Wound presentation of the right toe in the patient. **(A)** Before admission, the distal phalanx of the right big toe was blackened and numb, with purulent discharge visible at the wound margin. **(B)** Postoperative wound debridement of the right toe performed in the emergency department.).

The patient was a 72-year-old male farmer who was admitted on April 22, 2025, due to “swelling and pain of the right toe accompanied by limited mouth opening for more than 3 days.” Eight days prior to admission (April 14, 2025), the patient sustained a laceration to the right toe from iron wire while transplanting rice seedlings during farm work, resulting in an open wound with severe contamination. No debridement or disinfection was performed; the wound was merely wrapped with tissue paper, after which the patient continued working. Three days prior to admission (April 19, 2025), the distal phalanx of the right hallux became darkened and numb, with gradual enlargement of the wound and marked pain. These symptoms were accompanied by mild dysphagia, limited mouth opening, trismus, and paroxysmal dyspnea. The patient subsequently presented to a local hospital, where wound disinfection and dressing were performed. As tetanus infection could not be excluded, he was referred to the emergency department of our hospital (Figure 1). The patient has no history of colorectal cancer or other gastrointestinal malignancies, no diabetes, hypertension, chronic liver/kidney disease, or other underlying diseases, no history of surgery/chemotherapy, and no long-term use of broad-spectrum antibiotics. Heart rate was 102 beats/min, blood pressure was 160/70 mmHg, and oxygen saturation on room air was 94%. Marked trismus was present (interincisal distance < 1 cm), with facial muscle rigidity and generalized increased muscle tone; opisthotonos was absent, while neck stiffness was positive. The distal toes of the right foot show dry gangrene without gas production, accompanied by purulent discharge and localized skin temperature elevation; the dorsalis pedis pulse of the right foot was palpable. Pain was pronounced, and mobility of the right ankle joint was limited, though no obvious abnormalities in perfusion were observed. Pulmonary auscultation revealed scattered rales in both lungs. White blood cell count: 6.25 × 10^9^/L; procalcitonin: 1.317 ng/mL; high-sensitivity C-reactive protein: 65.69 mg/L; creatine kinase-MB: 0.81 μg/L; myoglobin: 78.44 μg/L; troponin: 0.03 μg/L; fibrinogen: 6.18 g/L; D-dimer: 1.38 mg/L; fibrin degradation products: 5.10 mg/L; the blood culture results were negative after 5 days of incubation under standard conditions. Chest imaging demonstrated small, indistinct patchy opacities in the dorsal subpleural regions of both lungs, consistent with aspiration pneumonia. Cranial CT revealed small ischemic lesions in the bilateral basal ganglia (possibly infarct foci), right maxillary sinusitis, and cerebral atrophy with widening of the subcranial spaces beneath the bilateral frontal bones (suggestive of a small amount of subdural effusion). The patient presented with foot trauma and urgent clinical symptoms and therefore was admitted. Because of the high cost of testing, the patient declined a head MRI and was unable to undergo brain MRI with DWI (diffusion-weighted imaging) and MRA (magnetic resonance angiography). Considering the clinical presentation and the CT findings of small ischemic lesions in the bilateral basal ganglia, a comprehensive assessment was made. The patient had no acute focal neurological deficits at any time and no prior history of cerebrovascular disease. Given the patient’s advanced age (72 years), the lesions are most consistent with chronic lacunar infarcts, which are common age-related degenerative changes and are unrelated to the current *C. septicum* infection.

**FIGURE 2:**
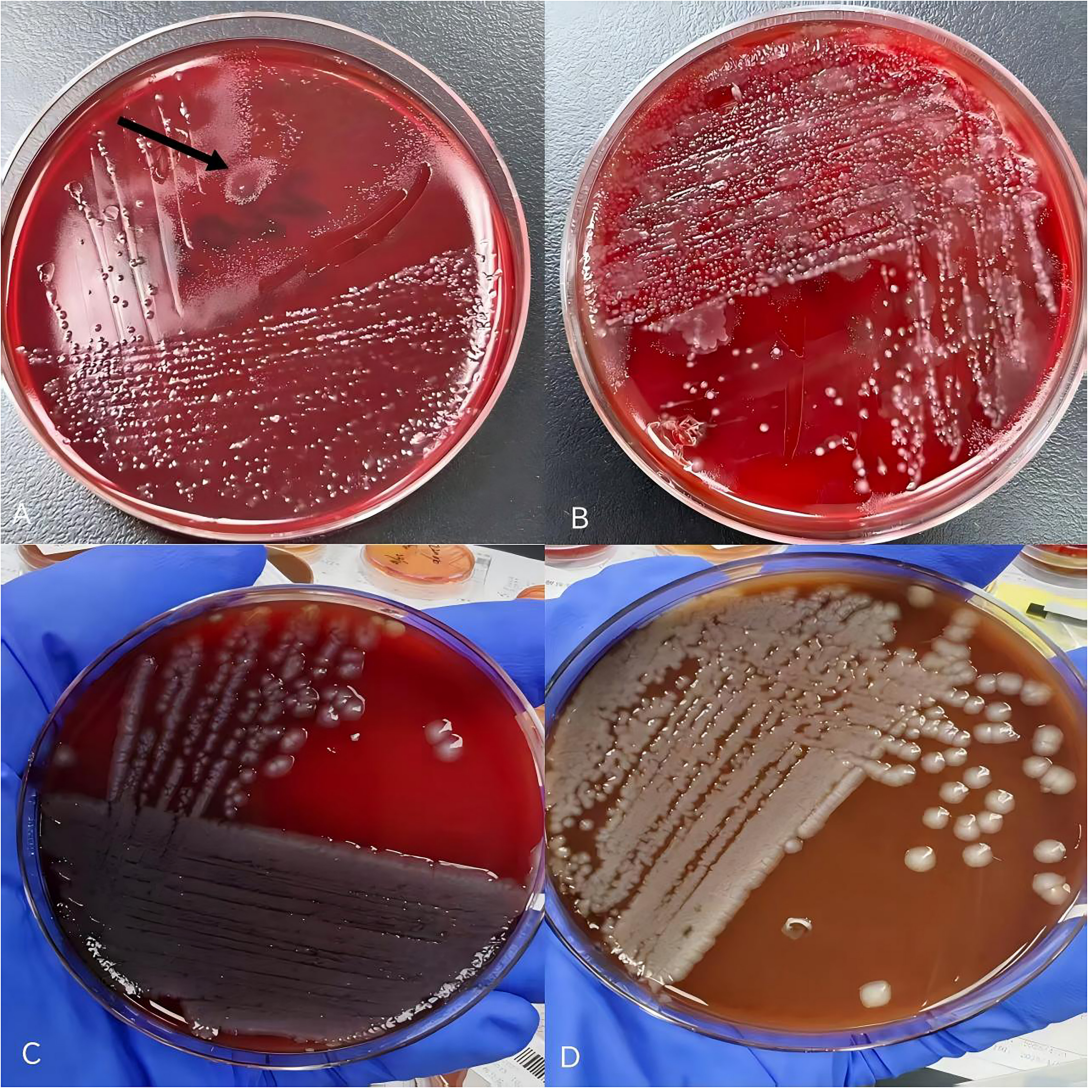
Colony morphology of secretion culture. **(A)** Colony morphology of secretion culture. The black arrow indicates the specific location of *Clostridium septicum.* Anaerobic culture on sheep blood agar for 24 h, feathery colonies with irregular edges, migratory growth, and foul odor. **(B)** Anaerobic culture on Columbia blood agar for 48 h, A small amount of *Clostridium septicum* remains present “tidal wave” like growth, A mixed growth pattern was observed for *Enterobacter cloacae* and *Clostridium septicum*. **(C)** After aerobic testing, petri dishes without *Clostridium septicum* growth, only showing *Enterobacter cloacae*. *Clostridium septicum* did not grow on blood agar plates. **(D)** After aerobic testing, petri dishes without *Clostridium septicum* growth, only showing *Enterobacter cloacae*. After aerobic testing, *Clostridium septicum* did not grow on chocolate agar plates.

**FIGURE 3:**
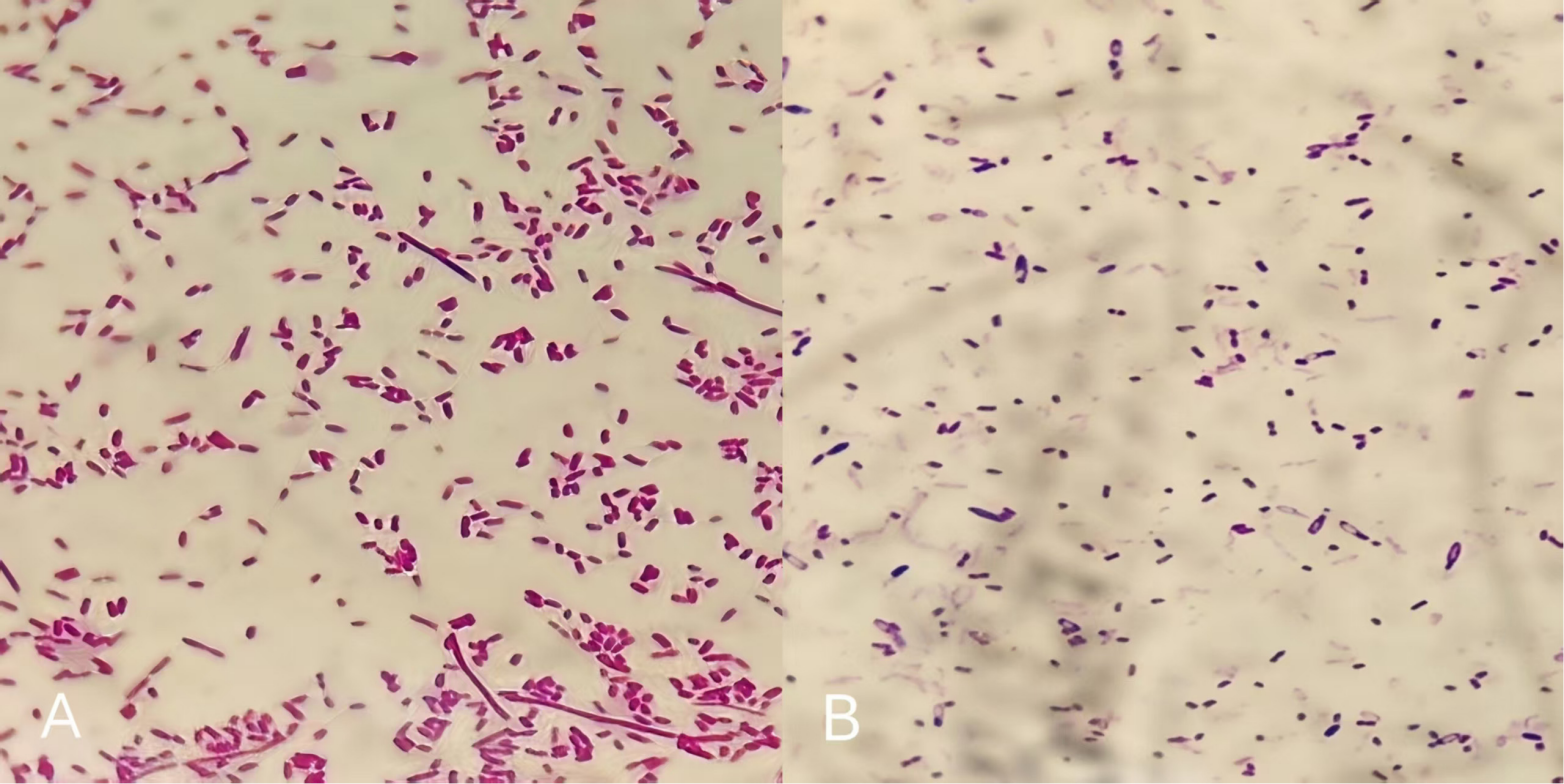
Gram staining of anaerobic culture (×1,000). **(A)** After 24 h of culture, thick bacilli, distributed singly, in pairs or in clusters, some filamentous. **(B)** After 48 h of culture, spores formed at one end of some bacteria, drumstick-shaped.

Bedside Gram staining of secretions revealed a large number of Gram-positive, thick, spore-forming bacilli with blunt ends, suggesting possible *Clostridium* species. Specimens were inoculated onto Columbia blood agar and cultured under anaerobic conditions at 35°C. After 24 h, gray-white membranous growth with irregular, feather-like colonies exhibiting migratory growth was observed (Figure 2A). After 48 h, a characteristic “tidal wave”–like growth pattern appeared (Figure 2B). Under aerobic culture for 24 h, gray-white, moist, smooth, non-hemolytic, medium-sized colonies were noted (Figures 2C,D). Smears prepared from 24-h anaerobic cultures showed purple, thick bacilli arranged singly, in pairs, or in clusters, with some organisms displaying filamentous forms (Figure 3A). After 48 h of culture, terminal spore formation was observed at one end of some bacilli, producing a drumstick-like appearance (Figure 3B). Identification by MALDI-TOF MS indicated *Clostridium septicum* and *Enterobacter cloacae* (with good reproducibility), and 16S rRNA gene sequencing results were concordant with the mass spectrometry findings. One week after admission, anaerobic culture and metagenomic next-generation sequencing (mNGS) were performed again on secretions obtained from the surgical wound. After 5 days, anaerobic cultures were reported as negative, and mNGS did not detect sequences of *Clostridium septicum* or *Clostridium tetani*.

**FIGURE 4:**
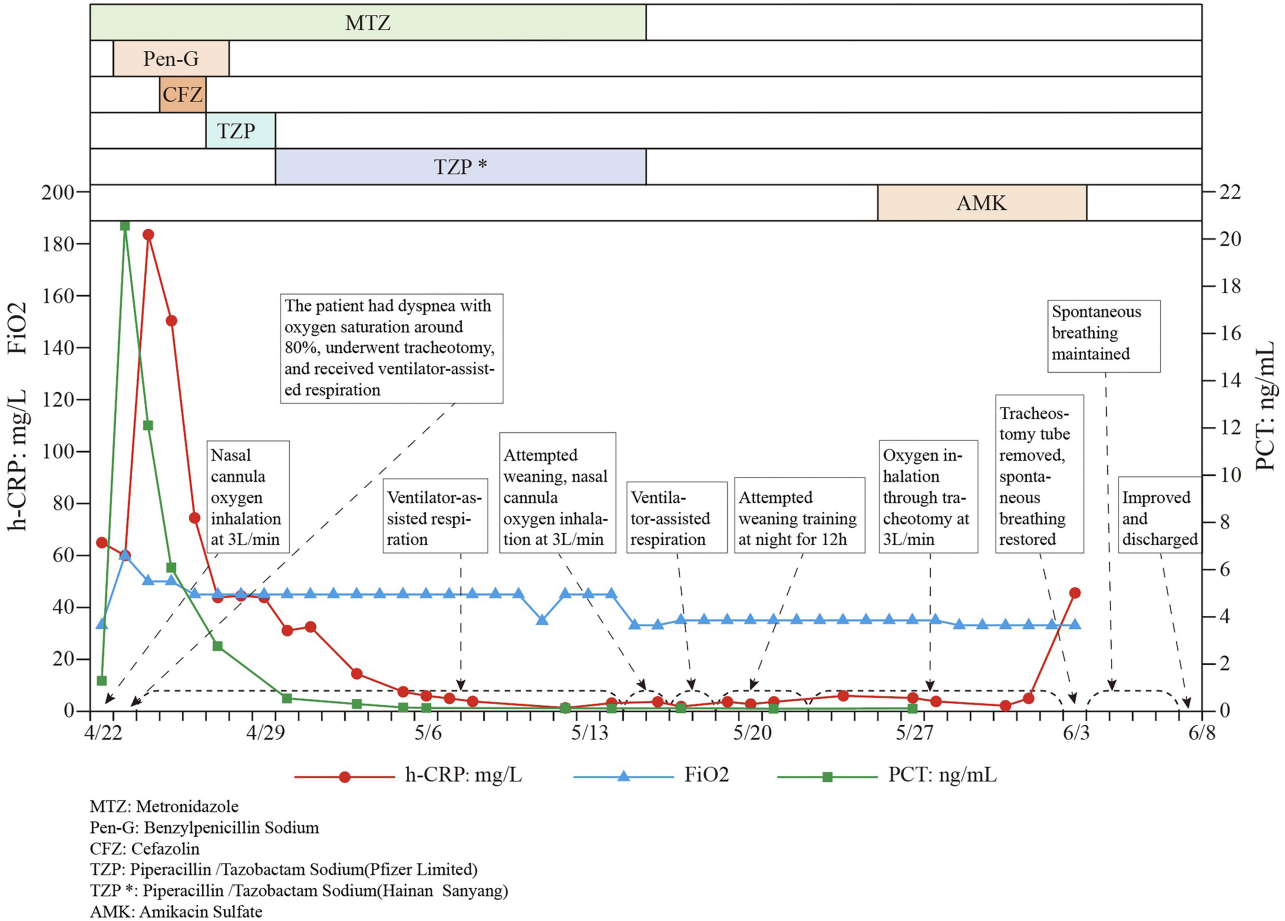
Diagnosis and treatment timeline of the patient. MTZ Metronidazole; Pen-G, Benzylpenicillin Sodium; CFZ, Cefazolin; TZP, Piperacillin/Tazobactam Sodium; TZP*, Piperacillin/Tazobactam Sodium; AMK, Amikacin Sulfate.

After the patient was admitted, medical staff immediately performed incision and debridement on the right toe, administered tetanus antitoxin, and collected wound secretions for microbiological testing. Empirical antimicrobial therapy with metronidazole combined with penicillin was initiated, supplemented by hyperbaric oxygen therapy. The patient was placed in a single, darkened room to minimize auditory and visual stimulation. Intravenous penicillin was administered at 2.4 million units per dose every 6 h, along with metronidazole 100 mL per dose every 6 h. Regular debridement was performed, with irrigation using hydrogen peroxide. In the early morning of the day following admission, the patient developed progressive respiratory distress, with oxygen saturation decreasing to approximately 80%, arterial pH of 7.22, and blood pressure of 98/67 mmHg. He was transferred to the intensive care unit, where prophylactic tracheostomy with placement of a silicone tracheal tube was performed, followed by mechanical ventilation (ASV mode; minute ventilation 100%, PEEP 5 cmH2O, FiO2 60%). Lactated Ringer’s solution (500 mL) was administered to correct metabolic acidosis, and the original antimicrobial regimen and tetanus antitoxin therapy were continued. On hospital day 3, the patient underwent “right hallux amputation plus debridement” under spinal anesthesia. The antimicrobial regimen was adjusted to piperacillin/tazobactam sodium 4.5 g per dose every 12 h, combined with penicillin and metronidazole at the original dosages. Tetanus antitoxin injections and daily hyperbaric oxygen therapy (2.5 atm for 90 min) were continued. In addition, nutritional support, prophylactic anticoagulation, magnesium sulfate for correction of autonomic dysfunction, and symptomatic treatments—including dexmedetomidine for sedation, remifentanil for analgesia, and vecuronium to reduce muscle tone—were provided. Pulmonary rehabilitation and muscle strength training were also implemented. Twenty days after tracheostomy, the patient’s condition stabilized, and he was transferred back to the general ward after successful nocturnal ventilator weaning trials. Thirty-five days postoperatively, mechanical ventilation was discontinued and replaced with oxygen delivery via the tracheostomy tube. The patient was discharged after rehabilitation on hospital day 48. The timeline of diagnosis and treatment is shown in Figure 4.

## 3 Discussion and literature review

Using the keywords “*Clostridium septicum*,” “severe infection,” “sepsis,” “tetanus,” and “gas gangrene,” literature searches were conducted in PubMed, China National Knowledge Infrastructure (CNKI), and Wanfang databases. The search time range is from 2021 to 2025. Case reports and clinical studies published in core Chinese journals and SCI-indexed journals were screened. Duplicate articles, reports with incomplete case information, and review articles were excluded. Ultimately, twelve relevant publications (including the present case) were included for analysis (Table 1). *Clostridium septicum* is a Gram-positive, anaerobic, spore-forming bacillus widely distributed in soil, sewage, and the intestinal tracts of animals. It is considered an opportunistic pathogen, and clinical infections are rare. Published reports indicate that high-risk populations include individuals with a history of trauma (particularly those with contaminated wounds that were not properly managed), diabetes mellitus, chronic kidney disease, immunocompromised states (e.g., malignancy, HIV infection), recent surgery or chemotherapy, and long-term use of broad-spectrum antibiotics leading to intestinal dysbiosis. Notably, approximately 50–60% of patients with positive blood cultures for *C. septicum* have concomitant gastrointestinal abnormalities, most commonly gastrointestinal malignancies. (10) Tumor-associated disruption of the mucosal barrier provides a portal of entry for bacterial invasion (11–13). The present patient had a clear history of trauma without standardized debridement, constituting a typical high-risk factor. *Clostridium septicum* infection is characterized by abrupt onset, rapid progression, and a mortality rate of up to 50%. Systemic manifestations predominantly include high fever, chills, and fatigue; in severe cases, septic shock and multiple organ dysfunction may develop rapidly. Localized infections may present as skin and soft tissue infections, necrotizing fasciitis, gas gangrene, lung abscesses, intra-abdominal infections, or septicemia, and some patients may develop migratory abscesses. Importantly, approximately 15% of patients may exhibit tetanus-like neuromuscular symptoms, such as trismus, lockjaw, generalized muscle rigidity, and respiratory distress, which can easily lead to misdiagnosis. The present case manifested these rare features, thereby expanding the clinical spectrum of *C. septicum* infection. Detailed analysis of pathogenesis indicates that the tetanus-like neuromuscular manifestations in this patient were not mediated by tetanospasmin from *Clostridium tetani* but were primarily attributable to the α-toxin of *C. septicum* and the local inflammatory response to infection. Prior studies show that *C. septicum* α-toxin (14), a pore-forming toxin, causes tissue necrosis and hemolysis and directly disrupts neuromuscular junction function by interfering with neurotransmitter release, thereby inducing sustained muscle contraction and spasm and producing clinical features that closely resemble tetanus. Concurrently, the wound’s local inflammatory response amplifies neuromuscular excitability and thus contributed to the patient’s trismus and restricted mouth opening. Nonspecific ischemic lesions on head CT should be interpreted in the context of the clinical presentation, and advanced imaging modalities such as DWI should be obtained when feasible to better characterize these lesions. Clinicians should remain alert to a potential association between *C. septicum* infection and cerebrovascular events.

**TABLE 1:** Summary of related domestic and international cases.

References	Patient characteristics (Age/Sex)	Underlying diseases/risk factors	Clinical manifestations	Diagnostic methods	Treatment regimen	Outcome
S. Bickerton et al. (11)	76 years/Female	Shoulder inflammation; no clear underlying disease; history of trauma	Necrotizing fasciitis; septic shock	Blood culture; Gram staining	Piperacillin–tazobactam + clindamycin	Death
Heino et al. (16)	37 years/Female	Previous surgical resection of pheochromocytoma; no underlying disease	Skin and soft tissue infection; necrotizing fasciitis	Pus culture; Gram staining	Meropenem + clindamycin + vancomycin + extensive debridement and excision + skin flap transplantation	Cure
Fejes et al. (18)	86 years / Female	B-cell lymphoma; rectal cancer	Necrotizing infection of the orbital region	Secretion culture; Gram staining	Ceftriaxone + metronidazole + amoxicillin–clavulanate + surgical intervention	Death
Tang Qinfang et al., (15)	60 years/Male	Pyogenic liver abscess	Gas gangrene; septicemia	Blood culture; pus culture; MALDI-TOF MS	Imipenem + linezolid + tinidazole + abscess drainage + CRRT	Death
Perl et al. (17)	62 years/Female	Breast cancer receiving chemotherapy; neutropenia	Left thigh cellulitis; multiple organ dysfunction	Secretion culture; Gram staining	Penicillin + clindamycin + surgical debridement	Cure
Sivasubramanian et al., (13)	83 years/Male	Cecal malignancy; history of biopsy	Migratory gas gangrene; septic shock	Blood culture; tissue culture	Imipenem + metronidazole + surgical resection of lesions	Death
Helmink et al. (12)	60 years / Male	Occult colorectal cancer	Progressive pneumocephalus; sepsis	Cerebrospinal fluid culture; 16S rRNA sequencing	Ceftriaxone + metronidazole + surgical drainage	Death
Fritzell et al., (19)	76 years/Male	Type-2 diabetes hypertension	Fever of 40 °C and radiating lower back pain	Secretion culture; Gram staining	IV benzylpenicillin combined with IV clindamycin +amoxicillin	Cure
LaGreca et al. (20)	71 years/	Type-2 diabetes, hyperlipidemia	Left forearm pain and swelling, Gas gangrene; septicemia	Clinical examination; X-ray; blood/tissue culture; colonoscopy	Surgical fasciotomy, proximal trans-humeral amputation	Survival; discharged to rehabilitation with functional prosthesis
Turban et al. (21)	45 years/Male	Overweight (BMI 28.9 kg/m^2^); refractory cardiac arrest with veno-arterial ECMO	Fulminant *C. septicum* gas gangrene; hemorrhagic bullae; septic shock; massive hemolysis	Blood culture; bullae fluid culture; MALDI-TOF; disc-diffusion test; pre-mortem CT	IV cefotaxime + amikacin; no surgical intervention	Death
Achebe et al. (22)	80 years/Female	Advanced myelodysplastic syndrome (MDS); transfusion-dependent; chemotherapy history	Necrotizing soft tissue infection; hemorrhagic bullae; *C. septicum* bacteremia; septic shock	Blood culture; X-ray; venous Doppler; CT; LRINEC score; laboratory testing	Delayed IV broad-spectrum antibiotics; palliative care; no surgical debridement	Death
Present case	72 years/Male	History of trauma (iron wire laceration without debridement)	Right toe gangrene; trismus; respiratory distress	Secretion culture; MALDI-TOF MS; 16S rRNA sequencing	Penicillin + metronidazole + piperacillin/tazobactam + toe amputation + tracheostomy + hyperbaric oxygen therapy	Cure

The diagnosis of *Clostridium septicum* infection relies primarily on etiological evidence. Blood cultures or cultures of local secretions constitute the traditional diagnostic standard; however, as an anaerobe, this organism requires prolonged incubation, typically 2–7 days, and culture positivity is influenced by factors such as timing of specimen collection and prior antibiotic exposure. Gram staining can rapidly suggest the presence of *Clostridium* species but cannot reliably distinguish *C. septicum* from other clostridia, including *Clostridium tetani*. Matrix-assisted laser desorption/ionization time-of-flight mass spectrometry (MALDI-TOF MS) enables rapid identification of cultured isolates, and when combined with 16S rRNA gene sequencing, allows for definitive diagnosis; the combined application of these methods improves diagnostic accuracy (11, 12, 15). Metagenomic next-generation sequencing (mNGS) permits rapid detection of pathogen nucleic acid sequences directly from clinical specimens without reliance on culture and is particularly useful for early, precise diagnosis of severe infections. mNGS played a limited role in differential diagnosis due to two key factors. First, the patient’s severely contaminated wound caused significant background interference during mNGS testing, making it challenging to obtain valid results. Second, *Clostridium septicum*, an obligate anaerobe and rare clinical pathogen, often leads to difficulties in accurate species matching during mNGS library construction and data analysis. The definitive diagnosis of *Clostridium septicum* in this case relied on traditional anaerobic culture, followed by precise identification using MALDI-TOF MS and 16S rRNA gene sequencing. This approach aligns with the clinical consensus that traditional anaerobic culture remains the gold standard for detecting rare anaerobic pathogens, whereas mNGS has inherent limitations in such cases. In addition to the aforementioned two reasons, the use of antibiotics prior to sampling can also lead to false-negative results. Which may explain the absence of *C. septicum* detection by mNGS in the later course of the present case. *Clostridium septicum* is highly susceptible to metronidazole, penicillin, carbapenems, and cefoxitin, while exhibiting relatively high resistance rates to gentamicin, fluoroquinolones, and macrolides. Clinical management therefore favors combination antimicrobial therapy. Empirical initial treatment commonly consists of metronidazole in combination with penicillin to ensure coverage of *Clostridium* species as well as potentially concomitant anaerobic and aerobic pathogens. Subsequent adjustment to broad-spectrum agents, such as piperacillin/tazobactam, should be guided by antimicrobial susceptibility testing. Beyond antimicrobial therapy, early and thorough surgical debridement with removal of necrotic tissue (e.g., toe amputation, as in the present case) is critical for infection control (16, 17). In patients who develop respiratory compromise, timely tracheostomy and mechanical ventilation are required to prevent respiratory failure (18). Hyperbaric oxygen therapy inhibits the growth and proliferation of anaerobic organisms and constitutes an important adjunctive treatment. Multidisciplinary collaboration—including emergency medicine, intensive care, surgery, and clinical microbiology—is essential for the management of severe infections and can significantly improve patient outcomes. Key determinants of prognosis in *Clostridium septicum* infection include early establishment of a definitive diagnosis, prompt initiation of targeted antimicrobial therapy, and the presence of underlying comorbidities and the severity of associated complications. According to the literature, delayed diagnosis or failure to administer effective antibiotics in a timely manner is associated with mortality rates exceeding 70%. In contrast, early precise diagnosis combined with standardized treatment—encompassing antimicrobial therapy, surgical debridement, and comprehensive supportive care—can reduce mortality to below 20%. Furthermore, systematic screening for and management of occult malignancies are crucial for improving long-term outcomes in affected patients.

As an opportunistic pathogen, *Clostridium septicum* infection is primarily associated with impairment of host mucosal barrier integrity or immunosuppression. Failure to perform standardized debridement after trauma constitutes a major precipitating factor, which is consistent with the disease background of the present case. Typical features of *C. septicum* infection include abrupt onset, rapid progression, and severe clinical course; notably, a subset of patients may present with tetanus-like neuromuscular manifestations, which readily leads to misdiagnosis. In the present case, the initial presentation of trismus and lockjaw prompted strong suspicion of tetanus. Key differential points from tetanus: We have elaborated on the key differentiation points, including incubation period, wound characteristics, pathogen identification, symptom distribution, and treatment response, and summarized these points in Table 2.

**TABLE 2:** Key differential points between *C. septicum* infection with tetanus-like symptoms and true *tetanus* caused by *C. tetani.*

Differential items	*C. septicum* infection with tetanus-like symptoms	Classical *Tetanus* caused by *C. tetani*	Supporting literature
Pathogenic toxin	Mediated by *C. septicum* α-toxin	Mediated by *C. tetani* tetanospasmin	Heino et al.; Achebe et al.; Chinese Physicians Association Emergency Physicians Branch Pempc, Beijing Emergency Medicine Society et al. (16, 22, 23)
Typical incubation period (post-trauma)	3–5 days (shorter onset, rapid progression)	7–10 days (classic incubation period, range: 3–21 days)	Perl et al.; Turban et al.; Chinese Physicians Association Emergency Physicians Branch Pempc, Beijing Emergency Medicine Society (17, 21, 23)
Wound characteristics	Obvious tissue gangrene, purulent discharge, and local inflammatory signs; occasional gas production in soft tissues.	Mild local inflammation without obvious tissue necrosis or gangrene; the wound may be healed when symptoms appear.	Bickerton et al.; Chinese Physicians Association Emergency Physicians Branch Pempc, Beijing Emergency Medicine Society (11, 23)
Core clinical manifestations	Predominant trismus + limited mouth opening; focal muscle rigidity; generalized rigidity and opisthotonos rare	Progressive generalized muscle rigidity; typical opisthotonos; persistent painful spasms; high risk of fatal respiratory spasm	Fritzell et al.; Achebe et al.; Chinese Physicians Association Emergency Physicians Branch Pempc, Beijing Emergency Medicine Society (19, 23)
Diagnostic gold standard	Positive *C. septicum* culture from wound/blood, confirmed by MALDI-TOF MS or 16S rRNA sequencing	Positive *C. tetani* culture or tetanospasmin detection in clinical specimens	Pia Heino et al.; Turban et al.; Chinese Physicians Association Emergency Physicians Branch Pempc, Beijing Emergency Medicine Society (16, 21, 23)
Core treatment response	Rapid symptom relief with targeted antimicrobial therapy + surgical debridement; tetanus antitoxin has limited effect	Slow symptom resolution; requires *tetanus* antitoxin to neutralize free toxin; spasms may persist for weeks	Perl et al.; Chinese Physicians Association Emergency Physicians Branch Pempc, Beijing Emergency Medicine Society (17, 23)
Common high-risk factors	Contaminated unmanaged trauma wounds, gastrointestinal malignancies, immunocompromise	Unmanaged trauma wounds (any severity); no obvious association with gastrointestinal malignancies/immunocompromise	Bickerton et al.; Achebe et al.; Chinese Physicians Association Emergency Physicians Branch Pempc, Beijing Emergency Medicine Society (11, 22, 23)
Imaging clues	May show soft tissue gas, myonecrosis, or abscess (CT/MRI); intracranial lesions rare	No specific imaging findings; rare intracranial complications (unrelated to direct infection)	Turban et al.; Fritzell et al.; *Chinese Physicians Association Emergency Physicians Branch Pempc, Beijing Emergency Medicine Society* (19, 21, 23)
Laboratory markers	Marked elevation of CRP, PCT; possible CK/MB/myoglobin elevation (rhabdomyolysis/myonecrosis)	Mild-moderate inflammatory elevation; CK elevation due to severe spasms (no myonecrosis)	Achebe et al.; Perl et al.; Chinese Physicians Association Emergency Physicians Branch Pempc, Beijing Emergency Medicine Society (17, 22, 23)

However, definitive diagnosis was ultimately established as mixed infection with *Clostridium septicum* and *Enterobacter cloacae* through the combined application of MALDI-TOF MS and 16S rRNA gene sequencing. *Enterobacter cloacae*, a member of the *Enterobacterales* order, is a common pathogen in wound infections and was readily cultured in this case. While its co-infection with *Clostridium septicum* increased the complexity of treatment, the severe clinical manifestations (e.g., tetanus-like symptoms, toe gangrene) were primarily driven by *C. septicum* due to its potent pathogenicity and toxin (α-toxin) effects, which exacerbated the infection. Targeted antibiotic therapy against *E. cloacae* was promptly administered based on antimicrobial susceptibility testing, ensuring effective coverage of this co-pathogen. Underscoring the necessity for vigilance in the differential diagnosis between these entities in clinical practice.

Therapeutically, antimicrobial regimens should provide coverage for anaerobic organisms as well as potential concomitant aerobic pathogens. Empirical initial therapy may consist of metronidazole combined with penicillin, with subsequent adjustment to broad-spectrum antibiotics (e.g., piperacillin/tazobactam) based on antimicrobial susceptibility testing. Concurrently, early and thorough debridement with removal of necrotic tissue is essential for infection control (16, 17). Hyperbaric oxygen therapy can inhibit anaerobic bacterial growth, while tracheostomy and mechanical ventilation are effective measures to prevent respiratory failure in patients with respiratory compromise (23). Multidisciplinary collaboration—integrating emergency medicine, intensive care, surgery, and clinical microbiology—is central to the management of severe infections, enabling rapid assessment, precise diagnosis, and comprehensive intervention, thereby significantly improving patient outcomes. In the present case, early etiological investigation facilitated definitive diagnosis, followed by timely initiation of antimicrobial therapy, surgical debridement with toe amputation, hyperbaric oxygen therapy, and tracheostomy, culminating in full recovery and discharge. This case provides practical reference for the diagnosis and management of similar rare infections (24). In conjunction with the literature review, we conclude that increasing clinical awareness of *C. septicum* infection—particularly the importance of differential diagnosis when neuromuscular symptoms arise after trauma; early initiation of etiological investigations, including Gram staining and culture, application of MALDI-TOF MS for identification, and 16S rRNA sequencing for difficult cases; formulation of individualized treatment strategies; and emphasis on multidisciplinary collaboration—are critical to reducing mortality associated with this severe infection. Given the rarity of *C. septicum* infection, further large-scale clinical studies are warranted to more clearly define optimal diagnostic algorithms and therapeutic strategies.

## Data Availability

The original contributions presented in this study are included in the article/supplementary material, further inquiries can be directed to the corresponding authors.

## References

[1] Aldape MJ Bayer CR Rice SN Bryant AE Stevens DL. Comparative efficacy of antibiotics in treating experimental *Clostridium septicum* infection. *Int J Antimicrob Agents.* (2018) 52:469–73. 10.1016/j.ijantimicag.2018.07.009 30012441 PMC6467573

[2] Amimoto K Sasaki Y Fukuyama S Tamura Y. Genetic variation and cross-reactivity of *Clostridium septicum* alpha-toxin. *Vet Microbiol.* (2006) 114:51–9. 10.1016/j.vetmic.2005.10.039 16337096

[3] Annapureddy N Agarwal SK Kanakadandi V Sabharwal MS Ammakkanavar N Simoes P et al. *Clostridium septicum* aortitis in a patient with extensive atheromatous disease of the aorta. *J Infect Chemother.* (2012) 18:948–50. 10.1007/s10156-012-0394-7 22410855

[4] Barnes C Gerstle JT Freedman MH Carcao MD. *Clostridium septicum* myonecrosis in congenital neutropenia. *Pediatrics.* (2004) 114:e757–60. 10.1542/peds.2004-0124 15574607

[5] Benamar S Cassir N Caputo A Cadoret F La Scola B. Complete genome sequence of *Clostridium septicum* strain CSUR P1044, isolated from the human gut microbiota. *Genome Announcements.* (2016) 4:e922–916. 10.1128/genomeA.00922-16 27609912 PMC5017217

[6] Ballard J Bryant A Stevens D Tweten RK. Purification and characterization of the lethal toxin (alpha-toxin) of *Clostridium septicum*. *Infect Immun.* (1992) 60:784–90. 10.1128/iai.60.3.784-790.1992 1541552 PMC257555

[7] Chakravorty A Awad MM Cheung JK Hiscox TJ Lyras D Rood JI. The pore-forming α-toxin from *Clostridium septicum* activates the MAPK pathway in a Ras-c-Raf-dependent and independent manner. *Toxins.* (2015) 7:516–34. 10.3390/toxins7020516 25675415 PMC4344638

[8] Curtis-Martínez C Sánchez-Guillén L. Emphysematous aortitis by *Clostridium septicum*: a rare and lethal complication of right colon cancer. *Eur J Vasc Endovasc Surg.* (2019) 57:509. 10.1016/j.ejvs.2018.12.022 30852054

[9] Eplinius F Hädrich C. Acute aortic dissection caused by *Clostridium septicum* aortitis. *Forensic Sci Int.* (2014) 244:e38–41. 10.1016/j.forsciint.2014.08.032 25242573

[10] Dahmus JD Kotler DL Kastenberg DM Kistler CA. The gut microbiome and colorectal cancer: a review of bacterial pathogenesis. *J Gastrointest Oncol.* (2018) 9:769–77. 10.21037/jgo.2018.04.07 30151274 PMC6087872

[11] Bickerton S Awopetu A Abood A Lee H Lane T Reid A. Atraumatic *Clostridium septicum* myonecrosis presenting as upper limb ischaemia in a patient with undiagnosed bowel cancer. *Ann R Coll Surg Engl.* (2022) 104:e95–7. 10.1308/rcsann.2021.0153 34825573 PMC10335302

[12] Helmink AJ Wahlig TA Fey PD Chen J Foster KW. 60-year-old male with rapidly progressive pneumocephalus caused by *Clostridium septicum* in the setting of an occult colonic adenocarcinoma. *BMC Infect Dis.* (2023) 23:189. 10.1186/s12879-023-08160-9 36997864 PMC10061804

[13] Sivasubramanian G. Rapidly progressive and fatal distant spontaneous gas gangrene due to *Clostridium septicum* after biopsy of malignant cecal mass. *IDCases.* (2021) 24:e01129. 10.1016/j.idcr.2021.e01129 34007786 PMC8111254

[14] Macha K Giede-Jeppe A Lücking H Coras R Huttner HB Held J. Ischaemic stroke and *Clostridium septicum* sepsis and meningitis in a patient with occult colon carcinoma - a case report and review of the literature. *BMC Neurol.* (2016) 16:239. 10.1186/s12883-016-0755-4 27881097 PMC5121982

[15] Tang Qinfang LR. A case of septic *Clostridium perfringens* gas gangrene and sepsis secondary to pyogenic liver abscess. *Chin J Infect Dis.* (2023) 41:220–2. 10.3760/cma.j.cn311365-20220811-00343

[16] Heino P Schepel V Malmi H Jahkola T. Gas gangrene caused by spontaneous *Clostridium septicum* infection: a case study. *Anaerobe.* (2023) 80:102719. 10.1016/j.anaerobe.2023.102719 36921887

[17] Perl T Jacquemai M Pedrazzi N Grobholz R Glaab R Conen A et al. Gas gangrene with *Clostridium septicum* in a neutropenic patient. *Infection.* (2025) 53:733–9. 10.1007/s15010-024-02401-y 39373951 PMC11971146

[18] Fejes I Dégi R Végh M. Clostridium septicum gas gangrene in the orbit: a case report. *Infection.* (2013) 41:267–70. 10.1007/s15010-012-0366-y 23203898

[19] Fritzell V Dwyer R Lindström D. Clostridium septicum causing aortic aneurysm, spondylodiscitis, and epidural abscess in an immunocompetent man. *BMC Infect Dis.* (2025) 25:1681. 10.1186/s12879-025-11852-z 41316033 PMC12670742

[20] LaGreca M Chen D Frick P Morway G Egger A Mayberry J. *Clostridium Septicum*: cause of gas gangrene in the upper extremity leading to proximal trans-humeral amputation, a review of *Clostridium septicum*. *J Orthop Case Rep.* (2024) 14:50–5. 10.13107/jocr.2024.v14.i11.4912 39524249 PMC11546050

[21] Turban A Joussellin V Piau C Cattoir V Launey Y Eustache G. Fatal *Clostridium septicum* gas gangrene complicating ECMO: case report and review of literature. *Access Microbiol.* (2024) 6:000825.v3. 10.1099/acmi.0.000825.v3 39104453 PMC11299951

[22] Achebe I Nwachukwu C Nzewi C. Spontaneous necrotizing soft tissue infection and fatal *Clostridium septicum* septicemia in myelodysplastic syndrome: a case report and comprehensive literature review. *Cureus.* (2025) 17:e84715. 10.7759/cureus.84715 40551931 PMC12183472

[23] Chinese Physicians Association Emergency Physicians Branch Pempc, Beijing Emergency Medicine Society Chinese expert consensus on the diagnosis, treatment, and prevention of adult tetanus in emergency medicine. *Chin J Crit Care Med.* (2025) 45:1013–21. 10.3969/j.issn.1002-1949.2025.12.001

[24] Panel CMGGDaTE. Consensus on diagnosis and treatment of clostridial myonecrosis (Gas Gangrene). *Zhejiang Med.* (2008) 30:664–6. 10.3969/j.issn.1006-2785.2008.06.067

